# Aqua­tris(1*H*-benzimidazole-κ*N* ^3^)(dichloro­acetato-κ*O*)copper(II) dichloro­acetate dihydrate

**DOI:** 10.1107/S1600536809038392

**Published:** 2009-09-26

**Authors:** Yu-Feng Li, Fang-Fang Jian

**Affiliations:** a Microscale Science Institute, Department of Chemistry and Chemical Engineering, Weifang University, Weifang 261061, People’s Republic of China; b Microscale Science Institute, Weifang University, Weifang 261061, People’s Republic of China

## Abstract

The title compound, [Cu(C_2_HCl_2_O_2_)(C_7_H_6_N_2_)_3_(H_2_O)]C_2_HCl_2_O_2_·2H_2_O, was prepared by reaction of copper(II) 2,2-dichloro­acetic acid and benzimidazole in ethanol solution. The compound shows a regular trigonal–bipyramidal stereochemistry. The Cu^II^ centre possesses a five-coordinated environment, coordinated by three N atoms from the three benzimidazole ligands and two O atoms, one from the dichloro­acetate ligand and the other from the coordinated water mol­ecule. The mol­ecular structure and packing are stabilized by O—H⋯O and N—H⋯O hydrogen bonds. The Cl atoms are disordered over two sites, with relative occupancies 0.67 (3) and 0.33 (3).

## Related literature

For background to penta-coordinated copper complexes, see: Tyagi *et al.* (1984 ▶). For a related compound, see: Barszcz *et al.* (2004 ▶).
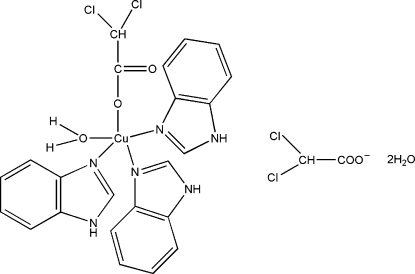


## Experimental

### 

#### Crystal data

[Cu(C_2_HCl_2_O_2_)(C_7_H_6_N_2_)_3_(H_2_O)]C_2_HCl_2_O_2_·2H_2_O
*M* *_r_* = 727.86
Monoclinic, 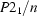

*a* = 9.6027 (16) Å
*b* = 8.6957 (15) Å
*c* = 37.799 (6) Å
β = 93.945 (3)°
*V* = 3148.8 (9) Å^3^
*Z* = 4
Mo *K*α radiation
μ = 1.09 mm^−1^
*T* = 293 K
0.23 × 0.20 × 0.18 mm

#### Data collection

Bruker SMART CCD area-detector diffractometer
Absorption correction: none
19614 measured reflections
7576 independent reflections
5174 reflections with *I* > 2σ(*I*)
*R*_int_ = 0.036

#### Refinement

*R*[*F* ^2^ > 2σ(*F* ^2^)] = 0.047
*wR*(*F* ^2^) = 0.122
*S* = 1.04
7576 reflections
488 parameters
10 restraints
H atoms treated by a mixture of independent and constrained refinement
Δρ_max_ = 1.00 e Å^−3^
Δρ_min_ = −0.59 e Å^−3^

### 

Data collection: *SMART* (Bruker, 1997 ▶); cell refinement: *SAINT* (Bruker, 1997 ▶); data reduction: *SAINT*; program(s) used to solve structure: *SHELXS97* (Sheldrick, 2008 ▶); program(s) used to refine structure: *SHELXL97* (Sheldrick, 2008 ▶); molecular graphics: *SHELXTL* (Sheldrick, 2008 ▶); software used to prepare material for publication: *SHELXTL*.

## Supplementary Material

Crystal structure: contains datablocks global, I. DOI: 10.1107/S1600536809038392/br2115sup1.cif

Structure factors: contains datablocks I. DOI: 10.1107/S1600536809038392/br2115Isup2.hkl

Additional supplementary materials:  crystallographic information; 3D view; checkCIF report

## Figures and Tables

**Table 1:** Hydrogen-bond geometry (Å, °)

*D*—H⋯*A*	*D*—H	H⋯*A*	*D*⋯*A*	*D*—H⋯*A*
O1*W*—H1*B*⋯O3^i^	0.808 (18)	1.955 (18)	2.763 (3)	178 (4)
O1*W*—H1*C*⋯O2*W*	0.802 (18)	2.18 (2)	2.949 (3)	162 (3)
N2—H2⋯O2*W*^ii^	0.820 (17)	2.08 (2)	2.873 (3)	162 (3)
N4—H4⋯O3*W*^iii^	0.856 (18)	2.00 (2)	2.842 (3)	166 (4)
N6—H6⋯O2^iv^	0.836 (18)	1.991 (19)	2.826 (3)	176 (3)
O2*W*—H2*B*⋯O3*W*	0.835 (18)	2.01 (2)	2.832 (3)	168 (4)
O2*W*—H2*C*⋯O4^iv^	0.810 (18)	2.080 (19)	2.886 (3)	172 (4)
O3*W*—H3*B*⋯O3	0.831 (17)	2.01 (2)	2.813 (4)	164 (3)
O3*W*—H3*C*⋯O4^i^	0.841 (17)	1.925 (18)	2.757 (3)	170 (4)
C15—H15*A*⋯O2	0.91 (3)	2.58 (3)	3.416 (4)	152 (3)

Symmetry codes: (i) 
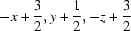
; (ii) 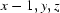; (iii) 
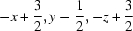
; (iv) 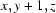.

**Fig. 1.**
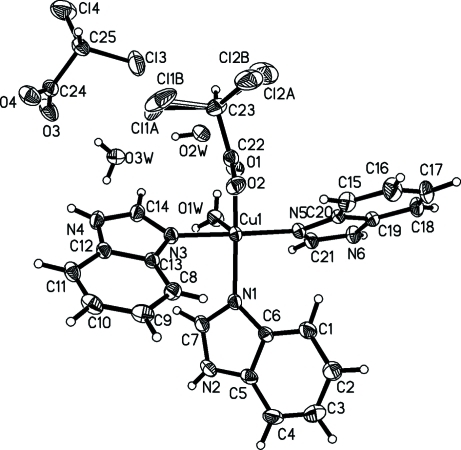
The molecular structure and atom-labeling scheme for (I), with displacement ellipsoids drawn at the 30% probability level.

## supplementary crystallographic information

### Comment

The penta-coordinated copper complexes have been attracting great interest for their diverse stereo and physicochemical properties (Tyagi *et al.*, 1984). Therefore the coordination chemistry of Cu(II) with ligands is of great interest. In this paper, we reported the synthesis and crystal structure of the title compound.

In the crystal structure of the title compound (Fig. 1), all the bond length and angle are in the normal range. (Barszcz *et al.*, 2004). The title compound consists of discrete monovalent complex cations, dichloroacetic acid anion and solvent water molecules. The dichloroacetic ions appear to be loosely held in lattice holes by Coulombic forces and by weak hydrogen bonds to the solvent water molecules. The interionic hydrogen bonds play an important role in the crystal packing and the stability of the complex. The Cl1 and Cl2 atoms are disordered.

### Experimental

Solid copper(II) 2,2-dicholoracetate, C_4_H_2_Cl_4_Cu_1_O_4_ 0.32 g (1 mmol) and benzimidazole 0.35 g (3 mmol) were added in 50 ml anhydrous alcohol under stirring. The mixture was refluxed for 5 h. The blue solution was filtered and the filtrate was left to stand undisturbed. Upon slow evaporation at room temperature, a blue crystalline solid appeared three days later and was separated by filtration. Determined by X-ray crystallography.

### Refinement

H atoms were positioned geometrically and allowed to ride on their parent atoms, with C—H distances of 0.93–0.96 Å, and with *U*_iso_(H) = 1.2*U*_eq_ of the parent atoms. The Cl1 atom and Cl2 atom are disordered over two sites, with relative occupancies 0.672 (34) and 0.328 (34).

### Crystal data

**Table:**

[Cu(C_2_HCl_2_O_2_)(C_7_H_6_N_2_)_3_(H_2_O)]C_2_HCl_2_O_2_·2H_2_O	*F*(000) = 1484
*M**_r_* = 727.86	*D*_x_ = 1.535 Mg m^−^^3^
Monoclinic, *P*2_1_/*n*	Mo *K*α radiation, λ = 0.71073 Å
Hall symbol: -P 2yn	Cell parameters from 2360 reflections
*a* = 9.6027 (16) Å	θ = 2.3–28.2°
*b* = 8.6957 (15) Å	µ = 1.09 mm^−^^1^
*c* = 37.799 (6) Å	*T* = 293 K
β = 93.945 (3)°	Block, blue
*V* = 3148.8 (9) Å^3^	0.23 × 0.20 × 0.18 mm
*Z* = 4	

### Data collection

**Table:**

Bruker SMART CCD area-detector diffractometer	5174 reflections with *I* > 2σ(*I*)
Radiation source: fine-focus sealed tube	*R*_int_ = 0.036
graphite	θ_max_ = 28.2°, θ_min_ = 2.2°
φ and ω scans	*h* = −12→10
19614 measured reflections	*k* = −11→11
7576 independent reflections	*l* = −34→50

### Refinement

**Table:**

Refinement on *F*^2^	Primary atom site location: structure-invariant direct methods
Least-squares matrix: full	Secondary atom site location: difference Fourier map
*R*[*F*^2^ > 2σ(*F*^2^)] = 0.047	Hydrogen site location: inferred from neighbouring sites
*wR*(*F*^2^) = 0.122	H atoms treated by a mixture of independent and constrained refinement
*S* = 1.04	*w* = 1/[σ^2^(*F*_o_^2^) + (0.0529*P*)^2^ + 1.2061*P*] where *P* = (*F*_o_^2^ + 2*F*_c_^2^)/3
7576 reflections	(Δ/σ)_max_ = 0.001
488 parameters	Δρ_max_ = 1.00 e Å^−^^3^
10 restraints	Δρ_min_ = −0.59 e Å^−^^3^

### Special details

**Table:**

Geometry. All e.s.d.'s (except the e.s.d. in the dihedral angle between two l.s. planes) are estimated using the full covariance matrix. The cell e.s.d.'s are taken into account individually in the estimation of e.s.d.'s in distances, angles and torsion angles; correlations between e.s.d.'s in cell parameters are only used when they are defined by crystal symmetry. An approximate (isotropic) treatment of cell e.s.d.'s is used for estimating e.s.d.'s involving l.s. planes.
Refinement. Refinement of *F*^2^ against ALL reflections. The weighted *R*-factor *wR* and goodness of fit *S* are based on *F*^2^, conventional *R*-factors *R* are based on *F*, with *F* set to zero for negative *F*^2^. The threshold expression of *F*^2^ > σ(*F*^2^) is used only for calculating *R*-factors(gt) *etc*. and is not relevant to the choice of reflections for refinement. *R*-factors based on *F*^2^ are statistically about twice as large as those based on *F*, and *R*- factors based on ALL data will be even larger.

### Fractional atomic coordinates and isotropic or equivalent isotropic displacement parameters (Å^2^)

**Table:**

	*x*	*y*	*z*	*U*_iso_*/*U*_eq_	Occ. (<1)
Cu1	0.55853 (3)	0.58911 (4)	0.621230 (8)	0.03147 (11)	
Cl1A	0.8686 (6)	0.1449 (6)	0.63210 (11)	0.0763 (11)	0.67 (3)
Cl2A	0.9346 (9)	0.2214 (14)	0.56111 (10)	0.087 (2)	0.67 (3)
Cl1B	0.8843 (14)	0.1269 (15)	0.6237 (7)	0.107 (4)	0.33 (3)
Cl2B	0.9719 (14)	0.298 (3)	0.5610 (2)	0.096 (3)	0.33 (3)
O1	0.7499 (2)	0.5103 (2)	0.61458 (6)	0.0418 (5)	
O2	0.6514 (2)	0.3248 (2)	0.58070 (6)	0.0461 (5)	
O1W	0.6458 (2)	0.7384 (3)	0.66834 (6)	0.0456 (5)	
H1B	0.619 (3)	0.741 (4)	0.6881 (6)	0.055*	
H1C	0.729 (2)	0.749 (4)	0.6710 (9)	0.055*	
N1	0.3528 (2)	0.6367 (3)	0.62332 (6)	0.0353 (5)	
N2	0.1489 (3)	0.6637 (3)	0.64701 (7)	0.0439 (6)	
H2	0.096 (3)	0.671 (4)	0.6629 (7)	0.044 (9)*	
N3	0.5337 (2)	0.4060 (3)	0.65180 (6)	0.0352 (5)	
N4	0.5672 (3)	0.2358 (3)	0.69497 (7)	0.0430 (6)	
H4	0.603 (3)	0.193 (4)	0.7139 (7)	0.066 (12)*	
N5	0.5864 (2)	0.7632 (3)	0.58822 (6)	0.0319 (5)	
N6	0.6065 (3)	1.0057 (3)	0.57153 (7)	0.0407 (6)	
H6	0.619 (4)	1.100 (2)	0.5752 (9)	0.058 (11)*	
C1	0.2615 (3)	0.6892 (4)	0.55998 (9)	0.0503 (8)	
H1A	0.353 (4)	0.666 (4)	0.5496 (9)	0.060*	
C2	0.1406 (4)	0.7264 (6)	0.53993 (11)	0.0736 (13)	
H2A	0.145 (4)	0.736 (5)	0.5136 (11)	0.088*	
C3	0.0141 (4)	0.7475 (6)	0.55558 (13)	0.0830 (14)	
H3A	−0.060 (3)	0.783 (5)	0.5426 (10)	0.100*	
C4	0.0025 (4)	0.7312 (5)	0.59114 (12)	0.0609 (10)	
H4A	−0.085 (4)	0.737 (4)	0.6013 (10)	0.073*	
C5	0.1233 (3)	0.6914 (3)	0.61125 (9)	0.0409 (7)	
C6	0.2516 (3)	0.6730 (3)	0.59624 (8)	0.0364 (6)	
C7	0.2861 (3)	0.6327 (4)	0.65275 (9)	0.0430 (7)	
H6A	0.330 (3)	0.608 (4)	0.6749 (9)	0.052*	
C8	0.3385 (3)	0.2603 (4)	0.61785 (8)	0.0408 (7)	
H8A	0.323 (3)	0.327 (4)	0.6009 (9)	0.049*	
C9	0.2590 (3)	0.1300 (4)	0.61945 (10)	0.0486 (8)	
H9A	0.188 (4)	0.110 (4)	0.6006 (9)	0.058*	
C10	0.2782 (4)	0.0263 (4)	0.64766 (10)	0.0515 (9)	
H10A	0.219 (4)	−0.060 (4)	0.6483 (9)	0.062*	
C11	0.3790 (4)	0.0449 (4)	0.67445 (9)	0.0459 (8)	
H11A	0.397 (3)	−0.027 (4)	0.6935 (9)	0.055*	
C12	0.4602 (3)	0.1785 (3)	0.67299 (7)	0.0347 (6)	
C13	0.4394 (3)	0.2852 (3)	0.64544 (7)	0.0330 (6)	
C14	0.6067 (3)	0.3692 (4)	0.68129 (8)	0.0413 (7)	
H14A	0.685 (3)	0.428 (4)	0.6910 (8)	0.050*	
C15	0.6404 (4)	0.6474 (4)	0.52901 (8)	0.0463 (8)	
H15A	0.634 (3)	0.546 (4)	0.5351 (9)	0.056*	
C16	0.6743 (4)	0.6869 (5)	0.49547 (9)	0.0633 (10)	
H16A	0.690 (4)	0.609 (5)	0.4791 (11)	0.076*	
C17	0.6901 (4)	0.8402 (5)	0.48565 (9)	0.0620 (10)	
H17A	0.717 (4)	0.858 (4)	0.4628 (10)	0.074*	
C18	0.6700 (4)	0.9586 (5)	0.50846 (9)	0.0509 (9)	
H18A	0.683 (4)	1.060 (4)	0.5054 (9)	0.061*	
C19	0.6356 (3)	0.9184 (3)	0.54269 (7)	0.0361 (6)	
C20	0.6221 (3)	0.7658 (3)	0.55289 (7)	0.0310 (6)	
C21	0.5786 (3)	0.9084 (4)	0.59758 (8)	0.0376 (6)	
H21A	0.559 (3)	0.941 (4)	0.6204 (8)	0.045*	
C22	0.7507 (3)	0.3848 (3)	0.59806 (8)	0.0397 (7)	
C23	0.8912 (4)	0.3007 (4)	0.60214 (9)	0.0517 (8)	
H23	0.963 (4)	0.356 (4)	0.6143 (9)	0.062*	
Cl3	1.08746 (13)	0.35382 (14)	0.69629 (3)	0.0815 (3)	
Cl4	1.26119 (11)	0.22307 (19)	0.75391 (3)	0.0951 (4)	
O3	0.9457 (3)	0.2538 (3)	0.76400 (7)	0.0684 (8)	
O4	0.9295 (3)	0.0293 (3)	0.73571 (7)	0.0609 (7)	
C24	0.9847 (3)	0.1547 (4)	0.74289 (8)	0.0446 (7)	
C25	1.1158 (3)	0.1918 (4)	0.72357 (9)	0.0481 (8)	
H25	1.136 (4)	0.111 (4)	0.7106 (9)	0.058*	
O2W	0.9420 (3)	0.7505 (3)	0.69464 (6)	0.0511 (6)	
H2B	0.924 (4)	0.685 (3)	0.7097 (8)	0.061*	
H2C	0.946 (4)	0.830 (3)	0.7056 (9)	0.061*	
O3W	0.8433 (2)	0.5492 (3)	0.74611 (6)	0.0478 (5)	
H3B	0.878 (4)	0.467 (3)	0.7505 (10)	0.057*	
H3C	0.762 (2)	0.530 (4)	0.7494 (10)	0.057*	

### Atomic displacement parameters (Å^2^)

**Table:**

	*U*^11^	*U*^22^	*U*^33^	*U*^12^	*U*^13^	*U*^23^
Cu1	0.03492 (19)	0.02875 (19)	0.03115 (18)	−0.00259 (14)	0.00518 (13)	0.00311 (14)
Cl1A	0.085 (2)	0.070 (2)	0.071 (3)	0.0185 (14)	−0.0159 (12)	0.0213 (11)
Cl2A	0.073 (2)	0.126 (4)	0.0618 (12)	0.044 (2)	0.0070 (12)	−0.0200 (18)
Cl1B	0.057 (4)	0.039 (3)	0.217 (10)	−0.003 (3)	−0.046 (5)	0.023 (5)
Cl2B	0.078 (4)	0.136 (9)	0.075 (3)	0.024 (5)	0.022 (3)	−0.024 (4)
O1	0.0390 (11)	0.0322 (11)	0.0552 (13)	−0.0025 (9)	0.0103 (9)	−0.0024 (10)
O2	0.0464 (12)	0.0340 (12)	0.0569 (13)	0.0046 (10)	−0.0030 (11)	−0.0003 (10)
O1W	0.0436 (12)	0.0581 (14)	0.0352 (11)	−0.0098 (11)	0.0024 (10)	−0.0060 (11)
N1	0.0392 (13)	0.0310 (13)	0.0363 (13)	−0.0004 (10)	0.0078 (10)	0.0054 (10)
N2	0.0443 (15)	0.0372 (15)	0.0529 (17)	−0.0025 (12)	0.0223 (13)	0.0032 (13)
N3	0.0381 (13)	0.0335 (13)	0.0338 (12)	−0.0063 (11)	−0.0001 (10)	0.0037 (10)
N4	0.0485 (15)	0.0453 (16)	0.0346 (14)	0.0015 (12)	−0.0015 (12)	0.0098 (12)
N5	0.0365 (12)	0.0289 (13)	0.0308 (12)	−0.0034 (10)	0.0065 (10)	0.0005 (10)
N6	0.0528 (16)	0.0266 (14)	0.0429 (14)	−0.0026 (12)	0.0058 (12)	0.0014 (12)
C1	0.0384 (17)	0.069 (2)	0.0433 (18)	−0.0134 (17)	0.0011 (14)	0.0042 (17)
C2	0.048 (2)	0.117 (4)	0.054 (2)	−0.020 (2)	−0.0085 (18)	0.020 (2)
C3	0.043 (2)	0.117 (4)	0.086 (3)	−0.012 (2)	−0.017 (2)	0.027 (3)
C4	0.0322 (18)	0.064 (2)	0.087 (3)	−0.0074 (16)	0.0059 (18)	0.013 (2)
C5	0.0346 (16)	0.0293 (16)	0.060 (2)	−0.0075 (13)	0.0093 (14)	0.0026 (14)
C6	0.0340 (15)	0.0343 (16)	0.0415 (16)	−0.0073 (12)	0.0054 (12)	0.0032 (13)
C7	0.0467 (18)	0.0417 (18)	0.0420 (17)	−0.0009 (14)	0.0138 (14)	0.0073 (14)
C8	0.0416 (17)	0.0400 (18)	0.0405 (17)	−0.0003 (14)	0.0002 (14)	−0.0006 (14)
C9	0.0414 (18)	0.048 (2)	0.057 (2)	−0.0067 (15)	0.0028 (15)	−0.0142 (16)
C10	0.052 (2)	0.0336 (18)	0.072 (2)	−0.0095 (15)	0.0215 (18)	−0.0107 (17)
C11	0.058 (2)	0.0296 (16)	0.053 (2)	0.0030 (15)	0.0230 (17)	0.0041 (14)
C12	0.0381 (15)	0.0333 (16)	0.0334 (15)	0.0015 (12)	0.0087 (12)	0.0015 (12)
C13	0.0348 (15)	0.0303 (15)	0.0345 (15)	−0.0005 (12)	0.0050 (12)	0.0019 (12)
C14	0.0432 (17)	0.0438 (19)	0.0360 (16)	−0.0045 (14)	−0.0034 (13)	0.0069 (13)
C15	0.059 (2)	0.0405 (18)	0.0392 (17)	0.0015 (16)	0.0032 (15)	−0.0056 (15)
C16	0.084 (3)	0.071 (3)	0.0350 (18)	0.008 (2)	0.0089 (18)	−0.0110 (18)
C17	0.076 (3)	0.082 (3)	0.0288 (17)	0.002 (2)	0.0124 (17)	0.0102 (18)
C18	0.057 (2)	0.053 (2)	0.0421 (18)	−0.0073 (17)	0.0058 (16)	0.0177 (17)
C19	0.0340 (14)	0.0395 (17)	0.0348 (15)	−0.0016 (13)	0.0032 (12)	0.0042 (13)
C20	0.0293 (13)	0.0349 (16)	0.0290 (13)	0.0001 (11)	0.0026 (11)	0.0011 (11)
C21	0.0438 (16)	0.0368 (16)	0.0331 (15)	0.0012 (13)	0.0092 (13)	0.0016 (13)
C22	0.0435 (17)	0.0339 (18)	0.0427 (17)	0.0025 (13)	0.0090 (14)	0.0057 (13)
C23	0.0475 (19)	0.052 (2)	0.055 (2)	0.0138 (16)	−0.0009 (16)	−0.0027 (17)
Cl3	0.1008 (8)	0.0806 (7)	0.0658 (6)	−0.0078 (6)	0.0254 (6)	0.0240 (6)
Cl4	0.0566 (6)	0.1605 (13)	0.0683 (7)	0.0004 (7)	0.0034 (5)	−0.0003 (7)
O3	0.0913 (19)	0.0503 (15)	0.0697 (16)	−0.0018 (13)	0.0500 (15)	−0.0059 (13)
O4	0.0587 (15)	0.0485 (15)	0.0781 (17)	−0.0107 (12)	0.0229 (13)	−0.0105 (13)
C24	0.0498 (18)	0.0414 (19)	0.0445 (18)	0.0068 (15)	0.0166 (15)	0.0040 (15)
C25	0.0519 (19)	0.049 (2)	0.0458 (18)	−0.0027 (16)	0.0185 (15)	−0.0044 (15)
O2W	0.0505 (13)	0.0561 (16)	0.0482 (14)	−0.0009 (12)	0.0135 (11)	−0.0052 (11)
O3W	0.0425 (13)	0.0501 (14)	0.0508 (13)	0.0010 (11)	0.0027 (11)	−0.0071 (11)

### Geometric parameters (Å, °)

**Table:**

Cu1—N3	1.991 (2)	C5—C6	1.400 (4)
Cu1—N5	1.992 (2)	C7—H6A	0.94 (3)
Cu1—O1	1.993 (2)	C8—C9	1.370 (4)
Cu1—N1	2.025 (2)	C8—C13	1.391 (4)
Cu1—O1W	2.314 (2)	C8—H8A	0.87 (3)
Cl1A—C23	1.789 (6)	C9—C10	1.399 (5)
Cl2A—C23	1.773 (5)	C9—H9A	0.97 (4)
Cl1B—C23	1.720 (11)	C10—C11	1.361 (5)
Cl2B—C23	1.784 (9)	C10—H10A	0.94 (4)
O1—C22	1.257 (3)	C11—C12	1.403 (4)
O2—C22	1.235 (4)	C11—H11A	0.96 (3)
O1W—H1B	0.808 (18)	C12—C13	1.399 (4)
O1W—H1C	0.803 (18)	C14—H14A	0.96 (3)
N1—C7	1.322 (4)	C15—C16	1.374 (5)
N1—C6	1.398 (4)	C15—C20	1.388 (4)
N2—C7	1.347 (4)	C15—H15A	0.91 (3)
N2—C5	1.378 (4)	C16—C17	1.395 (6)
N2—H2	0.820 (17)	C16—H16A	0.94 (4)
N3—C14	1.315 (4)	C17—C18	1.365 (6)
N3—C13	1.397 (4)	C17—H17A	0.93 (4)
N4—C14	1.336 (4)	C18—C19	1.401 (4)
N4—C12	1.370 (4)	C18—H18A	0.90 (4)
N4—H4	0.856 (18)	C19—C20	1.391 (4)
N5—C21	1.315 (4)	C21—H21A	0.94 (3)
N5—C20	1.401 (3)	C22—C23	1.533 (4)
N6—C21	1.339 (4)	C23—H23	0.93 (4)
N6—C19	1.373 (4)	Cl3—C25	1.756 (4)
N6—H6	0.835 (18)	Cl4—C25	1.766 (4)
C1—C2	1.380 (5)	O3—C24	1.249 (4)
C1—C6	1.388 (4)	O4—C24	1.235 (4)
C1—H1A	1.01 (3)	C24—C25	1.532 (4)
C2—C3	1.400 (6)	C25—H25	0.88 (3)
C2—H2A	1.00 (4)	O2W—H2B	0.835 (18)
C3—C4	1.364 (6)	O2W—H2C	0.809 (18)
C3—H3A	0.891 (19)	O3W—H3B	0.802 (18)
C4—C5	1.387 (5)	O3W—H3C	0.814 (18)
C4—H4A	0.95 (4)		
			
N3—Cu1—N5	176.38 (9)	C10—C11—C12	116.0 (3)
N3—Cu1—O1	86.92 (9)	C10—C11—H11A	124 (2)
N5—Cu1—O1	91.01 (9)	C12—C11—H11A	120 (2)
N3—Cu1—N1	89.13 (9)	N4—C12—C13	105.8 (2)
N5—Cu1—N1	92.43 (9)	N4—C12—C11	132.3 (3)
O1—Cu1—N1	170.17 (9)	C13—C12—C11	121.8 (3)
N3—Cu1—O1W	93.07 (9)	C8—C13—N3	131.0 (3)
N5—Cu1—O1W	89.91 (9)	C8—C13—C12	120.8 (3)
O1—Cu1—O1W	90.11 (9)	N3—C13—C12	108.2 (2)
N1—Cu1—O1W	99.09 (9)	N3—C14—N4	113.0 (3)
C22—O1—Cu1	113.46 (19)	N3—C14—H14A	123 (2)
Cu1—O1W—H1B	127 (3)	N4—C14—H14A	123.4 (19)
Cu1—O1W—H1C	118 (3)	C16—C15—C20	117.6 (3)
H1B—O1W—H1C	105 (4)	C16—C15—H15A	120 (2)
C7—N1—C6	105.6 (2)	C20—C15—H15A	122 (2)
C7—N1—Cu1	123.9 (2)	C15—C16—C17	121.4 (4)
C6—N1—Cu1	130.43 (18)	C15—C16—H16A	119 (2)
C7—N2—C5	107.4 (3)	C17—C16—H16A	119 (2)
C7—N2—H2	123 (2)	C18—C17—C16	122.0 (3)
C5—N2—H2	129 (2)	C18—C17—H17A	121 (3)
C14—N3—C13	105.3 (2)	C16—C17—H17A	117 (2)
C14—N3—Cu1	127.5 (2)	C17—C18—C19	116.6 (3)
C13—N3—Cu1	127.11 (19)	C17—C18—H18A	129 (2)
C14—N4—C12	107.6 (3)	C19—C18—H18A	114 (2)
C14—N4—H4	127 (3)	N6—C19—C20	106.2 (2)
C12—N4—H4	126 (3)	N6—C19—C18	132.0 (3)
C21—N5—C20	105.3 (2)	C20—C19—C18	121.8 (3)
C21—N5—Cu1	123.29 (19)	C15—C20—C19	120.6 (3)
C20—N5—Cu1	131.40 (18)	C15—C20—N5	131.1 (3)
C21—N6—C19	107.3 (3)	C19—C20—N5	108.2 (2)
C21—N6—H6	122 (2)	N5—C21—N6	113.0 (3)
C19—N6—H6	129 (2)	N5—C21—H21A	124 (2)
C2—C1—C6	117.0 (3)	N6—C21—H21A	123 (2)
C2—C1—H1A	123.8 (19)	O2—C22—O1	126.9 (3)
C6—C1—H1A	119.0 (19)	O2—C22—C23	119.6 (3)
C1—C2—C3	121.4 (4)	O1—C22—C23	113.5 (3)
C1—C2—H2A	118 (2)	C22—C23—Cl1B	113.9 (7)
C3—C2—H2A	120 (2)	C22—C23—Cl2A	110.8 (3)
C4—C3—C2	122.3 (4)	Cl1B—C23—Cl2A	95.2 (7)
C4—C3—H3A	117 (3)	C22—C23—Cl2B	110.6 (4)
C2—C3—H3A	120 (3)	Cl1B—C23—Cl2B	115.6 (5)
C3—C4—C5	116.4 (3)	Cl2A—C23—Cl2B	24.5 (5)
C3—C4—H4A	122 (2)	C22—C23—Cl1A	106.3 (3)
C5—C4—H4A	121 (2)	Cl1B—C23—Cl1A	12.7 (10)
N2—C5—C4	131.9 (3)	Cl2A—C23—Cl1A	107.8 (4)
N2—C5—C6	105.9 (3)	Cl2B—C23—Cl1A	128.3 (8)
C4—C5—C6	122.2 (3)	C22—C23—H23	115 (2)
C1—C6—N1	130.9 (3)	Cl1B—C23—H23	106 (2)
C1—C6—C5	120.7 (3)	Cl2A—C23—H23	115 (2)
N1—C6—C5	108.4 (2)	Cl2B—C23—H23	95 (2)
N1—C7—N2	112.6 (3)	Cl1A—C23—H23	101 (2)
N1—C7—H6A	123 (2)	O4—C24—O3	127.3 (3)
N2—C7—H6A	124 (2)	O4—C24—C25	115.8 (3)
C9—C8—C13	117.2 (3)	O3—C24—C25	116.9 (3)
C9—C8—H8A	121 (2)	C24—C25—Cl3	110.5 (2)
C13—C8—H8A	122 (2)	C24—C25—Cl4	111.2 (2)
C8—C9—C10	121.5 (3)	Cl3—C25—Cl4	109.9 (2)
C8—C9—H9A	119 (2)	C24—C25—H25	108 (2)
C10—C9—H9A	120 (2)	Cl3—C25—H25	110 (2)
C11—C10—C9	122.7 (3)	Cl4—C25—H25	107 (2)
C11—C10—H10A	118 (2)	H2B—O2W—H2C	104 (4)
C9—C10—H10A	119 (2)	H3B—O3W—H3C	100 (4)

### Hydrogen-bond geometry (Å, °)

**Table:**

*D*—H···*A*	*D*—H	H···*A*	*D*···*A*	*D*—H···*A*
O1W—H1B···O3^i^	0.81 (2)	1.96 (2)	2.763 (3)	178 (4)
O1W—H1C···O2W	0.80 (2)	2.18 (2)	2.949 (3)	162 (3)
N2—H2···O2W^ii^	0.82 (2)	2.08 (2)	2.873 (3)	162 (3)
N4—H4···O3W^iii^	0.86 (2)	2.00 (2)	2.842 (3)	166 (4)
N6—H6···O2^iv^	0.84 (2)	1.99 (2)	2.826 (3)	176 (3)
O2W—H2B···O3W	0.84 (2)	2.01 (2)	2.832 (3)	168 (4)
O2W—H2C···O4^iv^	0.81 (2)	2.08 (2)	2.886 (3)	172 (4)
O3W—H3B···O3	0.83 (2)	2.01 (2)	2.813 (4)	164 (3)
O3W—H3C···O4^i^	0.84 (2)	1.93 (2)	2.757 (3)	170 (4)
C15—H15A···O2	0.91 (3)	2.58 (3)	3.416 (4)	152 (3)

Symmetry codes: (i) −*x*+3/2, *y*+1/2, −*z*+3/2; (ii) *x*−1, *y*, *z*; (iii) −*x*+3/2, *y*−1/2, −*z*+3/2; (iv) *x*, *y*+1, *z*.
